# Supplementary material for: Extensive variation in the intelectin gene family in laboratory and wild mouse strains
Source: Sci Rep. 2021 Jul 30;11:15548. doi: 10.1038/s41598-021-94679-3 (PMC8324875; doi:10.1038/s41598-021-94679-3)
Supplement: Supplementary file 1 — Supplementary Information. [file 41598_2021_94679_MOESM1_ESM.pdf]

Supplementary Information

Extensive variation in the intelectin gene family in laboratory and wild mouse strains

Faisal Almalki (1*), Eric B. Nonnecke (2*), Patricia A. Castillo (2), Alex Bevin-Holder (1), Kristian K. Ullrich (3), Bo Lönnerdal (4), Linda Odenthal-Hesse (3), Charles L. Bevins (2**), Edward J. Hollox (1**)

Supplementary Figure 1 *ITLN1* and *ITLN2* gene expression patterns in humans

Supplementary Figure 2 High throughput sequencing strategy to measure relative expression abundance of individual mouse intelectins.

Supplementary Table 1 Relative mRNA abundance (%) of mouse intelectin paralogues in small intestine and colon of 129S2/SvPasCrl, as determine by high-throughput sequencing.

Supplementary Table 2 Presence of particular intelectin genes in mouse strains determined by PRT.

Supplementary Table 3 Mouse sequences used.

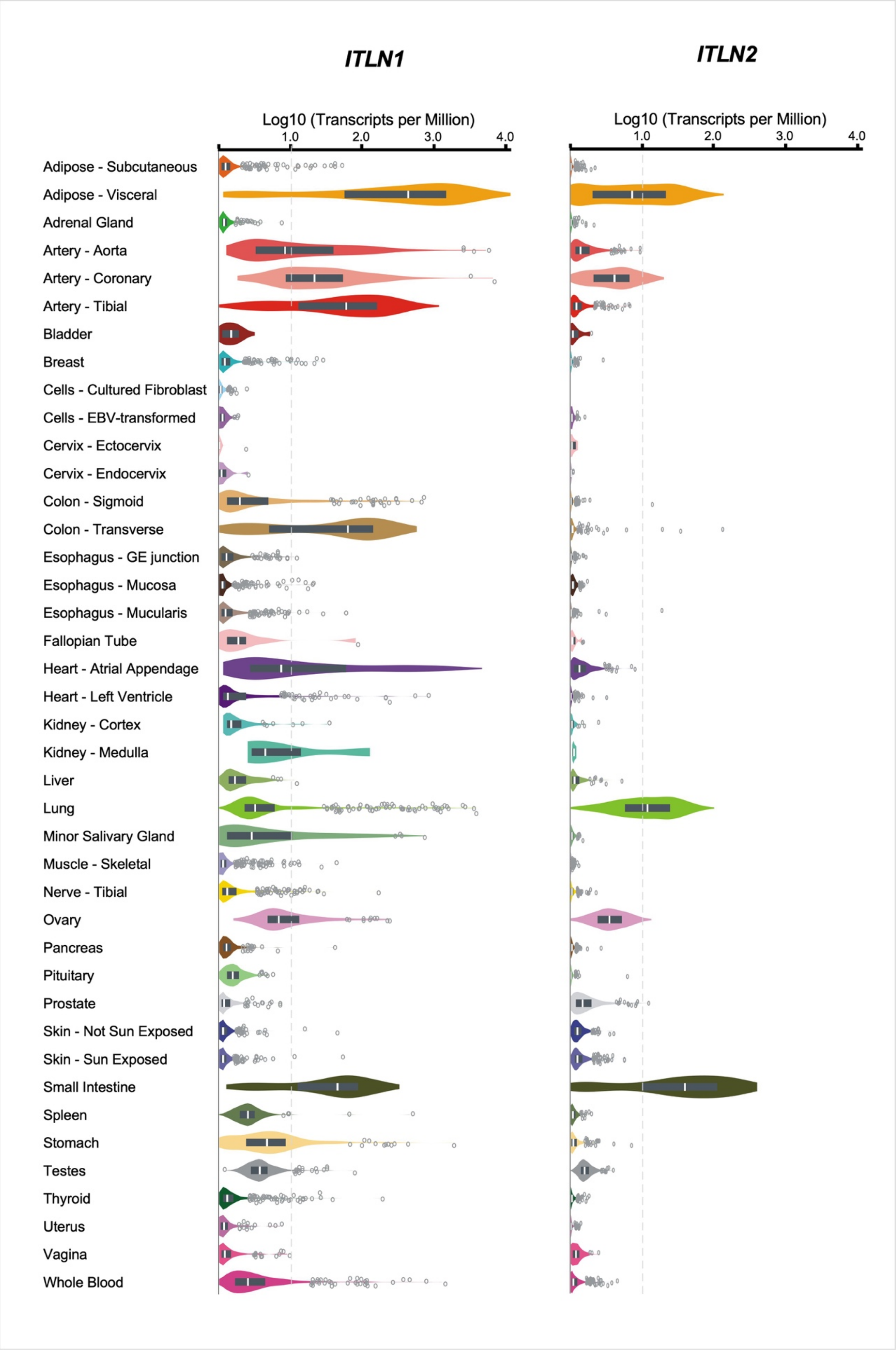

Supplementary Figure 1. ***ITLN1* and *ITLN2* gene expression patterns in humans.** Expression levels measured by RNAseq over a number of different human individuals for 53 tissues, ordered by median expression level. Data from Gtex database, plotted using GTEX browser. Data from 13 brain sections showed negligible expression and were omitted from the graph.

A.

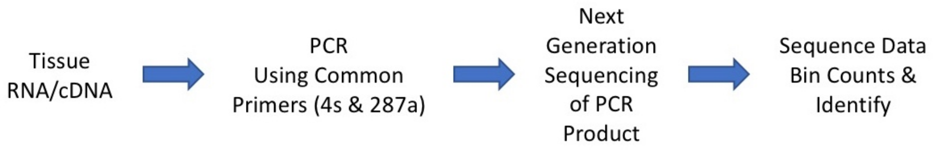

B.

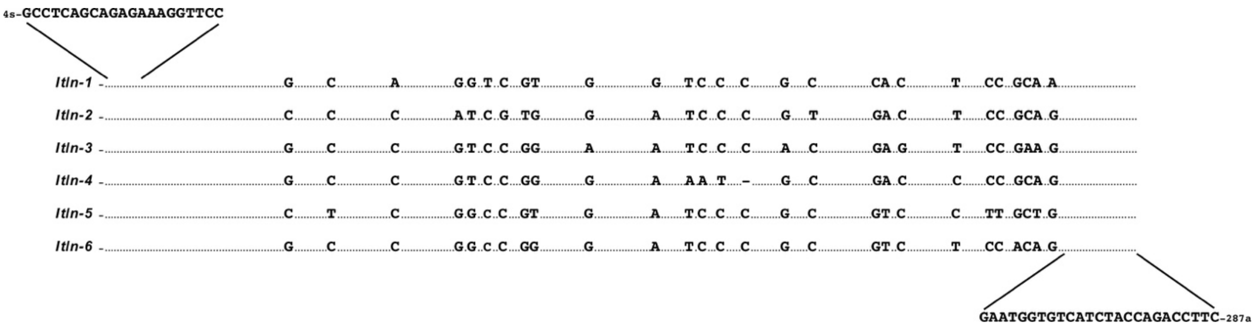

Supplementary Figure 2 High throughput sequencing strategy to measure relative expression abundance of individual mouse intelectins.

A. Outline of experimental strategy. B. Primers and ortholog sequence variations. Sequence alignment of PCR product (283 nt) showing nucleotides that differ among the six mouse intelectin genes.

Supplementary Table 1. Relative mRNA abundance (%) of mouse intelectin paralogues in small intestine and colon of 129S2/SvPasCrl, as determine by high-throughput sequencing.

Gene	Sequence Bin Count	
	Small Intestine	Colon
Itln1	112,357<br>(99.83%)	0
Itln2	246<br>(0.22%)	48<br>(0.06%)
Itln3	0	0
Itln4	0	0
Itln5	0	0
Itln6	168<br>(0.15%)	82,566<br>(99.94%)

Supplementary Table 2. Presence of particular intelectin genes in mouse strains determined by PRT.

P=present, A=absent, ND= not determined.

CRL = Charles River Laboratories, JL = Jackson Laboratories, TB = Taconic Biosciences

Subgenus, Species, & Subspecies	Strain/Substrain	*Vendor:ID*	*Itln1*	*Itln2*	*Itln4*	*Itln6*
Mus musculus* – mosaic: *domesticus/musculus	C57BL/6NCrl	CRL:574	P	A	A	A
	C57BL/6NTac	TB: B6	P	A	A	A
	C57BL/6NJ	JL: 005304	P	A	A	A
	C57BL/6J	JL: 000664	P	A	A	A
	C57BL/10J	JL: 000665	P	A	A	A
	C57BL/10SnJ	JL: 000666	P	A	A	A
	C57BLKS/J	JL: 000662	P	A	A	A
	C57L/J	JL: 000668	P	A	A	A
	C57BR/cdJ	JL: 000667	P	A	A	A
	C58/J	JL: 000669	P	A	A	A
	B6N-*Tyrc-Brd*/BrdCrCrl	CRL: 493	P	A	A	A
	C57BL/6NTac-$Tyr^{tm1Arte}$	TB: 11971	P	A	A	A

Supplementary Table 2 (continued)

	B6(Cg)-*Tyr*$^{c\text{-}2J}$/J	JL: 000058	P	A	A	A
	129S1/SvImJ	JL: 002448	P	P	P	P
	129S2/SvPasCrl	CRL: 476	P	P	P	P
	129S4/SvJaeJ	JL: 009104	P	P	P	P
	129S6/SvEvTac	TB: 129SVE	P	P	P	P
	129S8/SvEvNimrJ	JL: 012809	P	P	P	P
	129X1/SvJ	JL: 000691	P	P	P	P
	129P3/J	JL: 000689	P	P	P	P
	BALB/cAnNCrl	CRL: 547	P	P	P	P
	BALB/cAnNTac	TB: BALB	P	P	P	P
	BALB/cJ	JL: 000651	P	P	P	P
	BALB/cByJ	JL: 001026	P	P	P	P
	DBA/1J	JL: 000670	P	P	P	P
	DBA/2J	JL: 000671	P	P	P	P
	CBA/J	JL: 000659	P	P	P	P
	C3H/HeJ	JL: 000659	P	P	P	P
	A/J	JL: 000646	P	P	P	P
	AKR/J	JL: 000648	P	P	P	P
	FVB/NJ	JL: 001800	P	P	P	P

Supplementary Table 2 (continued)

	NOD/shiLtJ	JL: 001976	P	P	P	P
	SJL/J	JL: 000686	P	P	P	P
	SWR/J	JL: 000689	P	P	P	P
	CD-1 IGS	CRL: 022	P	P	P	P
	Crl:CFW(SW)	CRL: 024	P	P	P	P
	Crl:NIHBL(S)	CRL: 492	P	P	P	P
Mus musculus domesticus	LEWES/EiJ	JL: 002798	ND	ND	ND	ND
	PERC/EiJ	JL: 001307	ND	ND	ND	ND
	WSB/EiJ	JL: 001145	P	P	P	P
Mus musculus musculus	SKIVE/EiJ	JL: 001393	P	A	P	P
Mus musculus musculus	MOLF/EiJ	JL: 000550	P	A	P	P
	PWD/PhJ	JL: 004660	P	P	P	P
	PWK/PhJ	JL: 003715	P	P	P	P
	SF/CamEiJ	JL: 000280	P	P	P	P
Mus caroli	CAROLI/EiJ	JL: 000926	P	P	P	P
Mus musculus castaneus	CAST/EiJ	JL: 000735	P	A	A	P
Mus hortulanus	PANCEVO/EiJ	JL: 001384	P	A	P	P
Mus pahari	*Mus pahari*/EiJ	JL: 002655	ND	ND	ND	ND
Mus spretus	SPRET/EiJ	JL: 001146	P	A	P	P

Supplementary Table 3. Mouse sequences used.

Accession numbers for raw laboratory mouse strains WGS data that were sequenced by the Wellcome Trust Sanger institute. Accessed at the European Nucleotide Archive

Sample ID	Accession Number	Species
129S1/SvImJ	ERS076385	*Mus musculus*
A/J	ERS075416, ERS138733, ERS212195	*Mus musculus*
BALB/cJ	ERS076386	*Mus musculus*
C3H/HeJ	ERS076383	*Mus musculus*
C57BL/6NJ	ERS076384	*Mus musculus*
CAST/EiJ	ERS076381	*Mus musculus castaneus*
CBA/J	ERS076379	*Mus musculus*
DBA/2J	ERS075663	*Mus musculus*
FVB/NJ	ERP000687	*Mus musculus*
NOD/ShiLtJ	ERS076389	*Mus musculus*
NZB/B1NJ	ERS661331	*Mus musculus*
NZO/HlLtJ	ERS076387	*Mus musculus*
PWK/PhJ	ERS076378	*Mus musculus musculus*
SPRET/EiJ	ERS076388	*Mus spretus*
WSB/EiJ	ERS076380	*Mus musculus domesticus*
